# Notes on Preparations for the Sick

**Published:** 1911-12

**Authors:** 


### Notes on Preparations for the Sick.

**Adalin, a new sedative and hypnotic—Tablets, gr. v.**—THE BAYER CO., London.—Adalin, chemically bromo-di-ethyl-acetyl-urea, is a white powder, almost tasteless and odourless, nearly insoluble in cold water, soluble in hot water, the solution being of neutral reaction.

According to the dosage, adalin acts as a sedative or a hypnotic. Although it consists partly of bromine, its action is not due to the halogen content, as the liberation of the latter is too slow and too incomplete for ionisation. The action must be attributed to the whole molecule.

Many prescribers are unwilling to employ powerful hypnotics, and there are many cases in which it is inadvisable or unnecessary to employ them, although sleep is indispensable. For example, a patient may be unable to go to sleep on account of some external exciting cause producing a nervous condition, although once sleep has come it will continue; and another patient may waken soon after falling asleep, and be unable to go to sleep again. In such conditions adalin is of especial service, as its action may be described as that of diminishing abnormally excited conditions of the brain and spinal cord, and thus paving the way for physiological sleep.

We have found that adalin produces no unpleasant after-effects, and patients awake refreshed and invigorated; they do not feel drowsy nor do they have any headache or sense of fatigue. It may safely be taken daily for a lengthy period.

As a sedative, adalin is employed in many directions, viz. in chorea, exophthalmic goitre, neuroses of the heart, with tachycardia, neurasthenia and hysteria.

In dental practice adalin has been especially recommended. A dose taken before operation makes the ordeal more tolerable to a nervous patient, and another given afterwards has a soothing effect. When local or general anæsthesia is resorted to, it is found that less of the anæsthetising agent is required if adalin is previously given.

---

**Ferro-Sajodin.**—The Bayer Co., London.—The organic Iodide Sajodin is the calcium salt of mono-iodo-behenic acid, a tasteless and odourless powder containing 24.5 per cent. of Iodin and 4.1 per cent. of calcium.

The compound with iron is in the tablet form. One or more tablets may be taken three times a day after meals, chewed or crushed before taking. It forms a convenient method of administration of the last two drugs, iodine and iron, in cases of anæmia, arterio-sclerosis, and other conditions, when iodide and iron treatment is necessary.

The powder is insoluble in water, and it contains 5.6 per cent. of iron in combination with 24.5 per cent. of iodine. It is not likely to produce the symptoms of iodism, and the effect of the iodine continues for several days. The tablets are pleasantly flavoured with chocolate, and are readily taken by children.

---

**Palatinoid—Malt Diastase.**—Oppenheimer, Son & Co., London.—Each palatinoid contains the equivalent of one teaspoonful of the most active extract of malt.

---

**Gynoval.**—The Bayer Co., London.—This nerve sedative is put up in four-grain capsules, which may be taken one hour after meals three times a day.

It is chemically the iso-borneol-ester of iso-valerianic acid, derived from valerian. It is a colourless liquid, insoluble in water, but soluble in alcohol, with a much less pronounced smell and taste than the ordinary valerian preparations.

It is indicated in conditions such as hysteria, dysmenorrhœa, and other conditions associated with pregnancy and the climateric.

In cases of nervous insomnia two or three capsules taken at bedtime give good results.

---

**Iodex (IODine EXternally).**—MENLEY & JAMES, London.—Is an ointment containing resublimed iodine in a neutral base to the extent of 5 per cent. therapeutically free. It is non-irritating and non-staining, for although intensely black, the colour rapidly disappears when iodex is rubbed into the skin. Being neutral, it may be combined with various other drugs, such as methyl-salicylat, menthol, aristol, thymol, etc., so that it has a large range of utility in cases where iodine medication is desired.

Rapid penetration and marked activity are shown by the fact that iodine is readily traceable in the saliva and urine within an hour or two after application. Parasitic infections of the skin respond very readily to treatment by iodex applied three times daily.

---

**Formalets—Throat Tablets.**—E. S. L. & W. LTD., London and Liverpool.—These tablets, containing formaldehyde, are of the usual kind, intended to serve as an antiseptic germicide in all septic conditions of the mouth and throat. They should be used frequently and slowly dissolved in the mouth.

---

**Palol.**—PARKE, DAVIS & CO., London.—This tonic nutrient and restorative is an agreeably flavoured dark red fluid, one ounce of which is said to contain a cod liver oil extract, called Gaduol, from 250 minims of the oil, combined with malt extract and the glycerophosphates of calcium and sodium. Its uses are obvious, and it should give good results.

---

**Valerobromine, Legrand.**—DARRASSE FRÈRES, Paris.—This combination, bromo-valerianate of soda, is prepared in liquid form and in capsules.

The liquid is a concentrated solution of one-tenth part of the crystallised drug in an aromatic and sweetened diluent. Two to six teaspoonfuls may be given daily, or four to twelve capsules, according to the case. It should be a useful addition to our resources for anti-nervous medication.

---

**Huxley's Glycero-phosphates, with formates.** — ANGLO-AMERICAN PHARM. CO., Croydon.—The formates are believed to be muscle tonics, stimulating the muscular walls of the heart, intestines, etc. This combination, either with or without strychnine, should have a large range of utility as a muscular tonic in all depressed conditions.

---

**Tabloids—Xaxa and Caffeine.**—Burroughs, Wellcome & Co., London.—Each tabloid contains :—

Xaxa, gr. iv.
Caffeine, gr. i.

The combination should be useful in many rheumatic conditions associated with headache.

